# First Report of *Trypanosoma vivax* (Duttonella), *Babesia bovis* and *Babesia bigemina* DNA in Cattle from the Galapagos Islands, Ecuador, and Its Relationship with *Anaplasma marginale*

**DOI:** 10.3390/pathogens13100910

**Published:** 2024-10-18

**Authors:** María Augusta Chávez-Larrea, Cristina Cholota-Iza, Michelle Yugcha-Diaz, Jorge Ron-Román, Freddy Proaño-Pérez, Alicia Maya-Delgado, Jimmy Jumbo-Moreira, Armando Reyna-Bello, Claude Saegerman

**Affiliations:** 1 Research Unit of Epidemiology and Risk Analysis Applied to Veterinary Sciences (UREAR-ULiège), Fundamental and Applied Research for Animal and Health (FARAH) Center, Department of Infections and Parasitic Diseases, Faculty of Veterinary Medicine, University of Liège, 4000 Liège, Belgium; machavezlarrea@student.uliege.be (M.A.C.-L.); aliciafermaya@gmail.com (A.M.-D.); 2 Grupo de Investigación en Sanidad Animal y Humana (GISAH), Departamento de Ciencias de la Vida y de la Agricultura, Universidad de las Fuerzas Armadas-ESPE, P.O. Box 171-5-231, Sangolqui 171103, Ecuador; cecholota@espe.edu.ec (C.C.-I.); emyugcha@espe.edu.ec (M.Y.-D.); jwron@espe.edu.ec (J.R.-R.); fwproano@espe.edu.ec (F.P.-P.); jimmyjumbo93@gmail.com (J.J.-M.); 3 Instituto de Investigación en Zoonosis—CIZ, Universidad Central del Ecuador, Quito 170521, Ecuador; 4 Carrera de Ingeniería en Biotecnología, Departamento de Ciencias de la Vida y de la Agricultura, Universidad de las Fuerzas Armadas ESPE, P.O. Box 171-5-231, Sangolqui 171103, Ecuador

**Keywords:** trypanosomoses, *Trypanosoma vivax*, cattle, Galápagos, *Anaplasma marginale*, *Babesia bovis*, *Babesia bigemina*, Ecuador

## Abstract

Bovine trypanosomoses, caused by *Trypanosoma vivax*, is a disease present in African and South American countries. This haemoflagellate protozoan parasite, as well as *Anaplasma marginale* and *Babesia* spp., are microorganisms that have a blood tropism, mainly causing fever and anaemia, which reduces the productive capacity of dairy or meat farms. This study aimed to detect *T. vivax* and other blood parasites in bovine herds in the Galapagos Islands. A total of 170 blood samples from bovines in 19 farms on Santa Cruz Island (the most populated) were collected and analyzed using different PCR techniques: *Da*-PCR and *CatL*-PCR to detect *Trypanosoma vivax*, *CatL*-PCR to detect *Trypanosoma theileri*, *ESAG*-PCR to detect *Trypanosoma evansi*, *18S rRNA*-PCR to detect *Babesia* spp., *rap-1*-PCR to detect *Babesia bovis*, *hyp*-PCR to detect *Babesia bigemina*, and *msp5*-PCR to detect *A. marginale*. The prevalence of *T. vivax*, *B. bovis*, *B. bigemina*, and *A. marginale* was estimated as 14.7%, 11.2%, 14.7%, and 67.1%, respectively. In this study, the presence of four haemotropic agents was evidenced in 26.3% (5/19) of the farms. Coinfected cattle (*A. marginale*, *B. bovis* and *B. bigemina*) had significantly higher body temperatures compared to others (two-sample Wilcoxon rank-sum test; *p*-value = 0.047). The molecular techniques used in this study demonstrated the presence of *T. vivax* and *B. bovis* in cattle from Santa Cruz Island in the Galapagos for the first time. The study also investigates the relationship between *T. vivax, A. marginale* and *Babesia* spp., making a significant contribution to the field of veterinary medicine.

## 1. Introduction

Trypanosomoses caused by *Trypanosoma vivax* in cattle is a disease reported in all South American countries except Suriname, Chile, and Uruguay [1]. In South America, *T. vivax* can present as epizootic outbreaks with clinical signs such as anaemia, fever, anorexia, weight loss, drop in milk production, neurological disorders, loss of reproductive capacity, and abortions [2,3,4]. It is expected to find subclinical carriers in chronic stages of the disease [5].

Other species, such as *T. evansi* and *T. theileri,* that affect cattle have been reported in South America. In the case of *T. theileri,* it is a cosmopolitan protozoan considered non-pathogenic for cattle. However, it can be an opportunistic parasite during coinfections with other haemotropic agents such as *T. vivax*, *T. evansi*, *A. marginale* and *Babesia* spp. [6]. *Trypanosoma evansi* can naturally parasitize several domestic and wild mammals, where its pathogenicity is varied [7]. In South America, the disease in cattle can present asymptomatically, although a decrease in haematocrit has been evidenced without other apparent clinical signs associated with the disease [8].

Coinfections of *T. vivax* with other pathogens, such as *A. marginale* and *Babesia* spp., have been reported in bovine production systems in Continental Ecuador [9]. This coinfection, accompanied by high parasitaemia, destroys erythrocytes by different mechanisms and produces clinical signs such as anaemia, pale mucous membranes, weakness, anorexia, and lethargy, which makes diagnosis difficult [10,11].

Ticks are involved in transmitting the aforementioned haemotropic agent of *Babesia* spp., and *Rhipicephalus microplus* is the main vector in South America [7]. *A. marginale* transmission is also mechanical, through blood-sucking flies such as horseflies and contaminated fomites, as well as ticks [10,12]. However, *T. vivax* is exclusively transmitted by blood-sucking flies, such as tabanids and *Stomoxis calcitrans* [5].

In American tropical and subtropical areas, *A. marginale*, *Babesia* spp. and *Trypanosoma* spp. cause economic losses that are difficult to estimate. These losses can be related to a drop in milk production, weight loss, mortality, treatment and control [10], and animal marketing [11]. In Argentina, after an outbreak of *T. vivax* in a farm with 220 dairy cows, losses were estimated at 58.802 USD due to animal deaths, abortions, and low or absence of milk production [13]. In the Pantanal area of Brazil, with a population of about 11 million cattle, an outbreak of *T. vivax* was reported in 1995; losses were estimated at 4% of the value of the herd due to treatment, deaths of cattle, abortions, and loss of production [14]. On the African continent, losses were estimated at 5 million dollars per year, mainly due to low milk and meat production [15].

Species within the genus of Trypanosoma can affect a wide animal variety including insects, birds, fish, amphibians, reptiles and mammals [16]. In South America, *T. vivax* has been detected in cattle, goats, sheep, horses, buffaloes, and deer; however, in Africa, approximately 40 wild mammal species can carry out the parasite [1]. Recent studies in continental Ecuador, using molecular techniques, demonstrated the presence of *T. theileri* in cattle from slaughterhouses and the Amazon region [9,17]. Medina et al. (2017), in a study in the Pastaza province of the Amazon region, found a seroprevalence of 31.03% for *Trypanosoma* spp. and 65.5% for *A. marginale* [18]. Until now, the distribution and epidemiological aspects of *T. vivax* are unknown, although the first evidence of this protozoan was reported by Wells et al. (1977) through the indirect immunofluorescence technique, with a seroprevalence of 22.3% [19]. Later, their presence was confirmed using molecular techniques in an outbreak in the province of Manabí [20].

In the Galapagos Islands, an insular region of Ecuador with 19 islands and 200 islets and rocks, only four islands are populated (Isabela, Santa Cruz, San Cristóbal, and Floreana) in which agricultural activities are carried out [21]. The 2014 Agricultural Census shows Galapagos Islands have 10,100 cattle [22]. A study carried out by Rhea et al. (2023) in Floreana Island showed the highest production is poultry (90 birds in eight farms), pigs (26 pigs in six farms) and cattle (28 cattle in five farms) [23]. In the Galapagos Islands, approximately 37.5% of producers have cattle, the species of most significant economic importance [24].

In the Galapagos Islands, the presence of *T. vivax* in cattle has yet to be demonstrated. However, in this province, agents transmitted by vectors have already been reported in cattle and other animal species. Gioia et al. (2017) found *A. marginale* on the islands of Santa Cruz, San Cristóbal, and Isabela, also demonstrating a prevalence of 93.2% in cattle; the same study identified the presence of *A. marginale*, *B. bigemina*, and *Borrelia theileri* in ticks [25]. In other species, such as dogs, antibodies against *Ehrlichia* spp. and *Anaplasma* spp. were found on Santa Cruz Island [26]. Another study on 390 marine iguanas (*Amblyrhynchus cristatus*) carried out on eleven islands showed that 25% of the iguanas were infected by haemotropic agents of the genus *Hepatozoon* and/or *Hemolivia* (Apicomplexa: Eucoccidiorida) [27].

The evidence of *T. vivax* and *Babesia* spp. in cattle of the Galapagos Islands will allow the establishment of control plans on livestock farms monitoring the probability of threat to endemic and introduced animal species on the island. Therefore, this study aimed to detect *T. vivax*, *Babesia* spp. and other blood pathogens, as well as the risks of mixed infections, in the cattle production systems of Santa Cruz Island.

## 2. Materials and Methods

### 2.1. Study Area and Collection of Blood Samples

**Figure 1:**
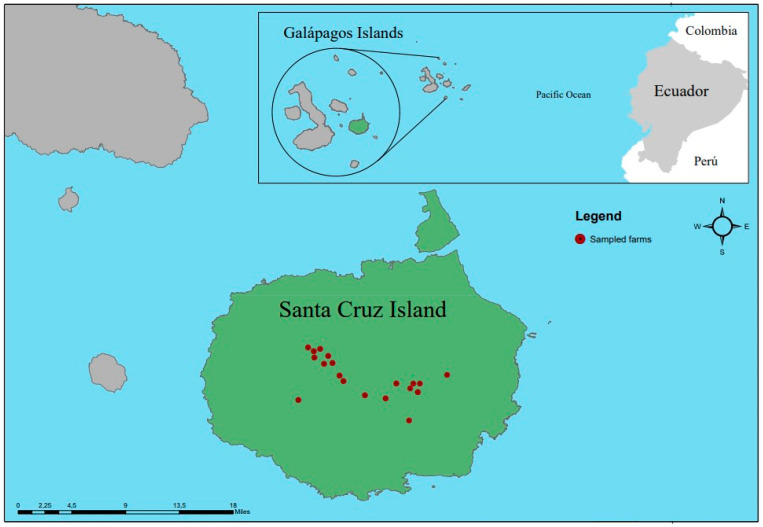
Location of the farms sampled on Santa Cruz Island, Galapagos Archipelago, Ecuador.

The insular province of Galapagos is located 972 km away from continental Ecuador and includes 761.844 hectares corresponding to the National Park (96.7%) and 26.356 hectares of populated area (3.3%) (Figure 1). Administratively, the territory is divided into three cantons: (1) Santa Cruz Island, made up of the Puerto Ayora, Bellavista, and Santa Rosa parishes; (2) San Cristóbal Island, with the parishes Puerto Baquerizo, Progreso and Santa María (Floreana Island), and (3) Isabela Island, with the parishes Puerto Villamil and Tomás de Berlanga. The temperature varies from 26 °C to 28 °C from January to April to less than 24 °C in the rest of the year [21].

This study was carried out on Santa Cruz Island in 2017 (Figure 1), located in the centre of the archipelago, which has an area of 986 km^2^ and a maximum altitude of 864 m above sea level [21].

The accuracy and precision of this study were ensured through the representativeness and size of the sample used. Sampling was carried out in two stages: (1) at the farm level and (2) at the animal level. For this purpose, the farms were categorized into small (less than 28 cattle), medium (between 29 and 60 cattle), and large (more than 61). There are approximately 755 UPAs in the Galapagos, 47% (355) of which are located in Santa Cruz [24], using a database facilitated by the ABG (Agency for Regulation and Control of Biosafety and Quarantine for Galapagos), 5.4% (19/355) were sampled on Santa Cruz Island. At the farm level, at least 10% of the animals were randomly sampled. In total, 170 cattle samples from Puerto Ayora, Bella Vista, and Santa Rosa in Santa Cruz Island were collected between February and June 2017. Blood samples were collected in tubes with EDTA by puncture of the coccygeal vein. Respecting the cold chain, the samples were first processed in the Fabricio Valverde laboratory of the Galapagos National Park and subsequently transported to Laboratorio de Biotecnología Animal at the Universidad de las Fuerzas Armadas ESPE (headquarters—Quito) for molecular tests analysis.

The zootechnical and sanitary-epidemiological information of the sampled cattle was collected through sampling and survey records. The sampled animals’ ages were divided into four categories: 0 to 9 months, 10 to 18 months, 19 to 36 months, and older than 36 months.

### 2.2. DNA Extraction

DNA extraction from cattle blood samples was performed using the Wizard^®^ Genomic DNA Purification Kit (Promega, Wisconsin, WI, USA), following the manufacturer’s instructions. The purity of the DNA was verified on 0.8% agarose gel electrophoresis and quantified by UV spectrophotometry using the Multiskan Sky High equipment (Thermo Fisher Scientific, Waltham, MA, USA).

### 2.3. Diagnostic of Haemotropic Agents by PCR

We performed a PCR end point to each primer pair (Table 1) to specifically detect the *T. vivax*, *T. evansi*, *T. theileri*, *B. bovis*, *B. bigemina*, and *A. marginale*. The diagnosis of *T. vivax* was made using ILO 1264 and ILO 1265 primers. In the case of *Babesia*, first, a PCR was run with PIRO A and PIRO B primers, which detected the presence of *Babesia* sp.; afterwards, the positive samples were run with *B. bovis* (BoF and BoR), and *B. bigemina* (BilA and BilB) specific primers.

GoTaq^®^ Green Master Mix 1X (Promega, Madison WI, USA), between 100–150 µg of DNA from each sample, and primer concentration specific for each primer were employed in the reaction mix. Primers concentration were 0.2 µM of ILO 1264 and ILO 1265, 0.25 µM of PIRO A and PIRO B, 0.3 µM of TthCatL and DTO155, 0.5 µM of TviCatL and DTO155, ESAG 6/7F and ESAG 6/7R, BoF and BoR, BilA and BilA, and 19A and 19B. Thermal cycler conditions were those established by the authors of the primers or modified in subsequent studies. These conditions are shown in Table 1; in some of the PCR, the hybridization temperatures were modified to improve results, mainly to eliminate nonspecific bands or increase the band intensity.

### 2.4. CatL-PCR to Detect T. vivax

The positive samples for ILO-PCR were run with *CatL*-PCR, using the TviCatL1 and DTO155 primers (Table 1) and the high-fidelity enzyme Platinum Taq DNA polymerase (Invitrogen, Waltham, MA, USA). The reaction conditions were those established in previous studies [20,28]. PCR products were purified using the Wizard^®^ SV Gel and Clean-Up System Kit (Promega, Madison, WI, USA) and sent to MACROGEN (Seoul, Republic of Korea) for Sanger sequencing.

### 2.5. Bioinformatic Analysis

We assembled the *CatL* sequences obtained using the MacVector 18.6 program, and the consensus sequences were deposited in GenBank (Accession numbers for CatL type: PP872140, PP872141, PP872142, PP872143). The similarity of consensus sequences obtained was analyzed with the BLAST tool. In the MEGA11 software, we built a maximum parsimony tree using the consensus sequences and those from GENBANK. The sequences used from *T. vivax* were reported in different countries in America and Africa.

### 2.6. Statistical Analysis

To identify possible explanatory variables associated with infection or coinfection with haemotropic agents, the molecular test distribution was analyzed based on certain variables collected per animal or sampled farm. The Chi-squared test was used for univariate analysis in animals (sex, age, breed). Fisher’s exact test was used for farm-level analysis (farm type, livestock movement, separate sick cattle, change needles, pasture rotation, presence of vectors, presence of other domestic animals, presence of dead animals last year, presence of urine with blood, muscle tremors). The two-sample Wilcoxon rank-sum test was used for temperature variable analysis. Age was categorized for the analyses as follows: (1) 0–9 months, (2) 10–18 months, (3) 19–36 months, (4) >36 months. Analyses were performed using Stata SE 14.2 (Stata Corp, College Station, TX, USA). The statistical significance threshold was set at a *p*-value ≤ 0.05.

## 3. Results

In 2017, 170 cattle samples were collected from 19 farms located on Santa Cruz Island in the Galapagos in Ecuador. The results showed that 23.5% (40/170) of blood samples came from Bella Vista, 8.2% (14/170) from Puerto Ayora, and 68.2% (116/170) from Santa Rosa. Regarding the zootechnical characteristics of cattle, 92.9% (158/170) were females, and 7.1% (12/170) were males. In total, 62.9% (107/170) belonged to the age category over 36 months, with an overall average age of 2.49 years (+/−0.79). The predominant bovine species was *Bos taurus*, with the following breeds: Simmental 45.9% (78/170), Brown Swiss 17.6% (30/170), Holstein 16.5% (28/170), 17.6% (30/170) were mixed breeds (*Bos taurus* and *Bos indicus*) and 2.4% (4/170) the race was not determined.

The prevalence of haemotropic agents molecularly identified at the farm level was as follows: *T. vivax* 42.1% (8/19), *B. bovis* 47.4% (9/19), *B. bigémina* 78.9% (15/19), *A. marginale* 100% (19/19). Regarding the prevalence per animal, no cases were observed for *T. vivax* and *B. bovis* in cattle sampled in Puerto Ayora. On the contrary, a high percentage (57.5%) of *A. marginale* was evident in Bellavista and the area in Puerto Ayora (71.4%) and Santa Rosa (69.8%). Prevalence at the animal level was 14.7% (25/170) to *T. vivax*. The presence of *T. evansi* and *T. theileri* could not be demonstrated in the cattle analyzed. However, the molecular tests revealed the presence of *B. bovis* at 11.2% (19/170) and *B. bigemina* at 14.7% (25/170). Table 2 presents the distribution of the farms and cattle sampled and the results of the molecular tests in the case of infections of haemotropic agents analyzed.

**Figure 2:**
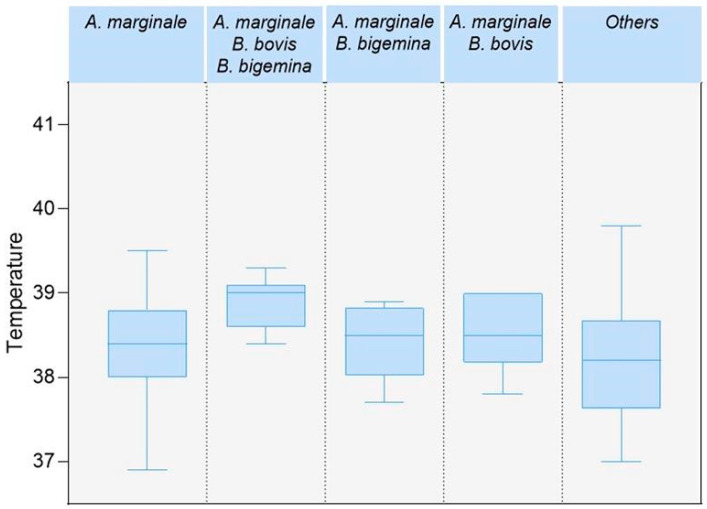
Box plot of the temperature distribution by type anaplasmosis and coinfections.

Among the haemotropic agents coinfections in the study, the most prevalent was *A. marginale* + *T. vivax* with 7.06% (12/170), while the lowest prevalence was *B. bovis + B. bigemina* and *T. vivax + B. bigemina + A. marginale* with 0.59% (1/170) (Table 3). An important fact to highlight is the presence of four haemotropic agents on 26.3% (5/19) of the farms in Table 4. In addition, a significantly higher body temperature in cattle (n = 6) with the coinfection including *A. marginale*, *B. bovis* and *B. bigemina* was observed (two-sample Wilcoxon rank-sum test; *p*-value = 0.047). Figure 2 shows the distribution of temperature in animals with anaplasmosis and coinfections.

Table 5 presents the distribution of cattle sampled and positive results of molecular diagnostic tests applied to identify haemotropic agents, considering the zootechnical parameters: sex, age, and breed. A higher prevalence of *B. bovis* (25%), *B. bigemina* (50%) and *A. marginale* (75%), was observed in males (n = 12). The highest prevalence was also found for all haemotropic agents in the category of age from 10 to 18 months (n = 16) as observed for *T. vivax* (25%), *B. bovis* (50%), *B. bigemina* (56.3%) and *A. marginale* (93.8%). About the breed, the highest prevalence could be observed in crossbreed cattle (n = 30) for *B. bovis* (16.7%), *B. bigemina* (26.7%), and *A. marginale* (93.3%), while *T. vivax* has a prevalence of 23.3% and was observed in Brown Swiss breed. The statistical analysis could not show a significant difference (Chi2 test; *p*-value > 0.05) in the distribution of positive samples depending on the zootechnical parameters.

Table 6 shows the distribution of positive results to the diagnostic tests, depending on the different sanitary management parameters observed in farms. The statistical analysis could not show a significant difference (Fischer’ exact test; *p*-value > 0.05) in the distribution of positive samples depending on the sanitary management parameters applied in farms. Regarding the general aspects of animal husbandry, the sampled farms were mostly mixed production (84.2%), the cattle were moved to other areas on the same island (57.9%), separated cattle when they were considered sick (84.2%), change needles when applying medications or vaccines (57.9%), and rotation of pastures (73.7%). In addition, 76.5% of farms reported the presence of ticks on cattle, and 68.4% reported their existence in other animals such as pigs, horses, poultry, goats, and dogs. The farms’ owners reported sudden deaths in cattle in the last year (31.6%), the presence of haematuria (10.5%), and muscle tremors (5.3%). Farms positive for *T. vivax* were significantly more associated with the presence of other domestic animal (Fischer’s exact test; *p*-value = 0.04).

**Figure 3:**
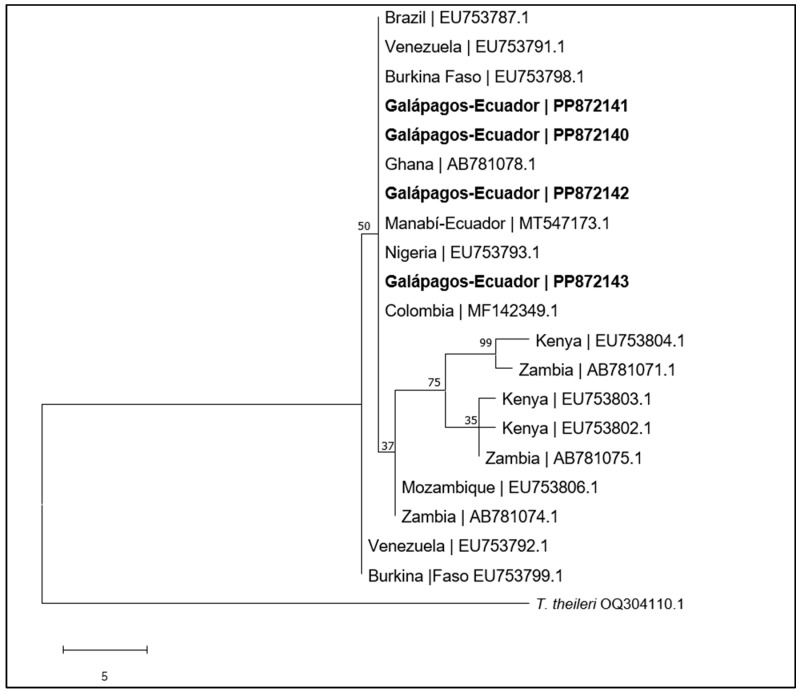
Maximum Parsimony tree of the Cathepsin gene of *Trypanosoma vivax* with sequences available in GenBank from different countries. Legend: PP872141, PP872140, PP872142 and PP872143: sequences obtained in this study from the Galapagos Islands, Ecuador. *T. theileri* sequence OQ304110.1 was used as an outgroup of the tree.

Positive samples (n = 4) for *CatL*-PCR were sequenced, showing a quality of over 95% when assembled. The sequences obtained were compared with other sequences (n = 16) found in the GenBank from 10 different countries. The dendrogram (Figure 2) shows a defined clade to *T. vivax* that confirms that the sequences of *T. vivax* from the Galapagos (PP872141, PP872140, PP872142 and PP872143) are joined with sequences from different countries in South America and Africa. A 100% similarity can be observed with the strains found in cattle from Ecuador, Brazil, Venezuela, Colombia, Ghana, Burkina Faso and Nigeria. The dendrogram yielded a consistency index, retention index, and composite index of 0.812500, 0.900000, and 0.857143 (0.731250), respectively. The *T. theileri* sequence (OQ304110.1) was used as an outgroup of the tree (Figure 3).

## 4. Discussion

### 4.1. Trypanosoma vivax

This study demonstrated the presence of *T. vivax* in the province of Galapagos for the first time, with a prevalence of 14.7% in cattle. This finding was made possible by the use of a PCR, which has a high sensitivity and is considered the selected test for the detection of active infections to diagnose bovine trypanosomoses [42]. An analytical test of sensitivity and specificity of five primers showed that ILO 1264/1265 primers had the best results [28]. Another molecular marker was *CatL* gen, which reports a high sensitivity and specificity and has been widely used to study genetic diversity, allowing the comparison of isolates obtained in Galapagos with those from South America and Africa [2,4,30,43]. Our finding would imply the endemic status of *T. vivax* on Santa Cruz Island, but *T. evansi* and *T. theileri* were not found in the analyzed samples.

Dendrogram analysis of the isolates of *T. vivax* from the Galapagos Islands revealed a similarity to those found in continental Ecuador, and they are closely related to those reported in Brazil, Venezuela, Colombia, Burkina Faso, Ghana and Nigeria (Figure 3). These findings suggest that in the Galapagos, *T. vivax* came from the continent, possibly from Ecuador itself, as has been found in other studies in South America, which indicate that the origin of this haemoflagellate is native to Africa [4,30,44,45].

Cattle (*Bos taurus*) were introduced to the Galapagos Islands during their colonization from mainland Ecuador, which began in 1832 with Floreana Island, followed by San Cristóbal in 1869 and Isabela in 1895. Santa Cruz Island was inhabited in the 1920s, and cattle arrived on the island after 1923 [46]. It is estimated that *T. vivax* was introduced into South America around 1830 through the importation of zebu cattle originating from Senegal, which entered mainly French Guiana and the French West Indies and dispersed throughout the rest of the continent [47]. Several mobilizations of cattle were carried out to the Galapagos Islands from different regions of continental Ecuador and between islands starting from colonization until 1999 when the Inspection and Quarantine System for Galapagos (SICGAL) was created to prevent the entry of exogenous species to the islands [21], this mobility probably favoured the entry of *T. vivax* and other haemotropic agents to the Galapagos.

The prevalence of *T. vivax* in this study was similar to the report in continental Ecuador by Chavez-Larrea et al. (2021) in the Quito slaughterhouse (13.3%) but higher than the prevalence in the province of Santo Domingo de los Tsáchilas (3.7%) using *CatL*-PCR [9]. The reported prevalence of *T. vivax* is concerning in endemic areas of South America, such as Brazil (state of Pará), where *T. vivax* infection rates detected by *CatL*-PCR were 24.6% [43]. However, it is higher than those found in the Caribbean and Orinoquia regions in Colombia, where the recorded prevalence was 0.2% [6] and 8.84% in the state of Goiás [3].

### 4.2. Anaplasma marginale

In this study, the presence of *A. marginale* was also evidenced in cattle of Santa Cruz Island, which was present in all the farms analyzed, with a prevalence within the herd of 67.1%. However, this prevalence is lower than that found by Gioia et al. (2018) in cattle from the Isabela, Santa Cruz and San Cristobal islands (93.2%) [25]. In continental Ecuador, it was reported that in the province of Zamora Chinchipe, the prevalence of A. marginale was 63.8% [48]. It was also lower than that described by Tana-Hernandez et al., of 86.1%, in a sampling carried out in the center of the country, specifically in the province of Santo Domingo de los Tsáchilas [40].

### 4.3. Babesia spp.

Although the presence of *B. bigemina* was reported on the islands through the analysis of a pool of ticks from Santa Cruz Island [25], the present work also allowed for the first report of *B. bovis* (11.2%) at the level of animals sampled on Santa Cruz Island, and confirm the presence of *B. bigemina* (14.7%) in cattle. Through *18S rRNA*-PCR, the presence of *Babesia* spp. was determined in 20% of the sampled cattle.

These haemotropic agents are present in bovine production systems and are considered endemic in South America, where prevalences of *A. marginale* and *Babesia* spp. have been reported in 48.9% and 39.8%, respectively [1]. In Colombia, molecular tests revealed prevalences of 59.3%, 31.5%, and 13.8% for *A. marginale*, *B. bigemina,* and *B. bovis*, respectively [6]. In another study in the south of Minas Gerais in Brazil, a 100% seroprevalence was evident for *A. marginale* and *B. bovis* [49].

### 4.4. Coinfections

Coinfections of haemotropic agents were also evaluated in this study, determining that the most common was *T. vivax* +*A. marginale* with 7.1 % and *B. bigemina* + *A. marginale* with 6.5%, this finding has been previously reported in South America according to Fetene et al. (2021), 26.1% of cattle had *A. marginale* + *Babesia* spp. coinfections [1]. In Colombia, coinfection infections were detected (*A. marginale* + *Babesia* spp. + *Trypanosoma* spp.), with a presence of 53.9% in the cattle analyzed [6]. In continental Ecuador, coinfections were demonstrated in cattle: 18.1% had double infection (*A. marginale* + *T. theileri*), and 6% had triple infection: *A. marginale* + *B. bovis* + *B. bigemina* [9].

This study demonstrated a significant correlation between animals infected with triple coinfection (*T. vivax*, *A. marginale*, *Babesia* spp.) and hyperthermia, a characteristic clinical sign of the three diseases during parasitaemia in the acute stage of infections [5,11,12]. Studies have shown that *T. vivax* causes immunosuppression; therefore, clinical signs intensify mainly when concomitant diseases exist [50]. Coinfections and the pathogenicity of the causal agent, host susceptibility, and stress influence the presence of clinical signs associated with these haemotropic agents [2,3,47,51]. In the case of *Babesia* spp., it has been shown that the development of clinical manifestations in cattle could be a delayed, inadequate, or insufficient immune response due to poor adaptation between *Babesia* spp. species and their vertebrate hosts [52].

The high prevalence observed in Galapagos for *A. marginale* and *Babesia* spp. could be associated with the transmission routes of agents. In our study, 76.5% of farms reported the presence of ticks on livestock, probably *R. microplus* species, which has been reported in the Galapagos Islands, and also observed *A. marginale* and *B. bigemina* in these ticks [25], as well as in a research conducted in mainland Ecuador, which reported a 60% of *A. marginale* in *R. microplus* ticks, and a 6% prevalence of *A. marginale* in *Amblyomma cajennense* ticks [53]. Thus, *R. microplus* is an important vector due to its wide regional distribution.

On the other hand, 23% of the farms sampled in Santa Cruz claimed to observe blood-sucking flies, although in this study, the species of flies were not identified. It is possible they can be Tabanidae flies since these have been reported previously in Santa Cruz [54]. Tabanids’ presence is associated with the prevalence and distribution of trypanosomoses, which have already been described in regions of America [6,15]. Iatrogenic transmission cannot be ruled out for *T. vivax* and *A. marginale* since 47.4% of the farms stated that they do not change the needle when administering vaccines or medications to cattle. This route has been associated with the presence of some outbreaks of *T. vivax* in Brazil [3,4] and transmission of *A. marginale* [55].

In the sampled farms, the presence of other domestic animals was evidenced as a risk factor for *T. vivax*. Some of them can harbour the parasite. This has been reported in different studies in South America, where *T. vivax* has been detected in goats, sheep, horses, buffalo, and deer [1]. In the Galapagos Islands, several species of domestic animals apart from cattle were introduced during colonization. These species are found in wildlife conditions with the effort to reduce or eliminate their presence in the Islands, such as feral cats (*Felis silvestris* catus), feral dogs (*Canis lupus* familiaris), feral donkeys (*Equus asinus*), feral horses (*E. caballus*), feral pig (*Sus scrofa*), feral goat (*Capra hircus*) and Domestic sheep (*Ovis aries*) among others [46]. Although there is no record of the number of times these species were imported from the continent to the Islands during the colonization, *T. vivax* likely entered with ruminant species, which could be reservoirs. The dispersion of these haemotropic agents in the Islands may be motivated by the mobility of local cattle; this could also be an essential factor related to the ticks’ introduction in new areas where susceptible vertebrate host species are found [56]. In this context, the presence of wildlife and introduced animals in the Galapagos Islands can be an essential source of vector-transmitted infectious diseases, which has previously been evidenced by Pike et al. (2020), who found protozoans of the trypanosomatid family in the introduced parasitic fly *Philornis downi* that affects passerine birds [57].

Similarly, mobility in cattle has been associated with *T. vivax* outbreaks [2,4,44] in free areas of the parasite. Although this study was carried out on one of the four islands where cattle exist, in the Galapagos, the mobility of cattle between areas within each island should be improved in future analyses of these haemotropic agents.

The most populated island in the Galapagos is Santa Cruz, which covers 47% of the 755 agricultural productive units (APU) in the island province. Cattle are destined for meat production (61%) and dairy (49%), and mixed breeds are the most representative (80%). It is reported that in recent years, the Simmental breed has been introduced via artificial insemination for the genetic improvement of livestock [21].

Our study showed a difference in the distribution of positive cattle to *Babesia* spp. depending on the breed. In the Holstein breed, the occurrence was 7.1% (2/38). It has been reported that both *Bos taurus* and *Bos indicus* are susceptible to *T. vivax* in Latin America [35], which can cause serious illness with infections by *Babesia* spp. and *A. marginale* [11], observing a possible resistance in pure individuals of *Bos indicus* that depends on the virulence of the agent [58]. Resistance to *B. bovis* has also been found in crosses of Hereford and Aberdeen Angus cattle (*Bos taurus*), finding the phenotypic frequency of 27.9%, showing in these animals tolerance to parasitism and low percentage of corpuscular volume (PCV) [59]. On the other hand, *Bos indicus* breeds resist ticks; some so-called Criollo breeds of Iberian origin distributed in South and Central America are also more resistant to *R. microplus* [60].

This study did not demonstrate a significant difference in the presentation of positive results depending on age for both *T. vivax* and *A. marginale*. However, it has been shown that the prevalence of *A. marginale* can increase with age, with mortality rates ranging from 50 to 60% in adults [10]. Further, there was a difference between the lower number of positive results for *Babesia* spp. depending on age over 36 months 11.2% (12/107). This has been shown in the case of *B. bovis*; animals over 36 months are less susceptible to infections by this parasite in Mexico [50]. The majority of cattle that tested positive for *T. vivax* (25%), *Babesia* spp. (62.3%) and *A. marginale* (93.3%) were found in the 10 to 18-month category. In a study in the El Carmen area, a coastal area of the territory of Ecuador, 42.18% of the asymptomatic positive cattle for *Babesia* spp. belonged to the age group of 10 to 18 months [20]. In regions where ticks are abundant, like in the Galapagos Islands, young cattle exposed to haemotropic agents are more resistant to infection due to concomitant immunity [61].

## 5. Conclusions

The application of molecular techniques allowed the first demonstration of the presence of *T. vivax, B. bovis* and *B. bigemina* in cattle on Santa Cruz Island, a province of the Galapagos in Ecuador. In addition, a high prevalence of other haemotropic agents was found, such as *A. marginale* and *Babesia* spp., which may suggest an endemicity of these diseases on the island. Moreover, cattle with signs such as hyperthermia were associated with triple haemotropic coinfection. No *T. evansi* DNA was detected in the analyzed samples, which corresponds to the absence of this species in cattle in continental Ecuador. It was also found that *T. vivax* infections were associated with other animal species on the farms, which suggests the importance of monitoring them since they can be possible reservoirs of several parasites on the island. These findings clarify epidemiological aspects of these diseases on Santa Cruz Island by generating updated scientific information, creating the basis for future control strategies formulation. These results could also be extrapolated and evaluated on the remaining islands of the archipelago where livestock activity exists. Therefore, livestock practices must consider a global epidemiological perspective (an island equal to a continent) and a conservationist perspective since islands host unique wild animal species worldwide.

## Figures and Tables

**Table 1:** Primer information used in this study.

Organism	Target Gen	Primers (Sequence 5′-3′)	Size (bp)	Thermocycler Conditions					References
				1 Cycle	35 Cycles			1 Cycle	
				ID	D	H	E	FE	
*T. vivax*	*Diagnostic antigen (Da)*	ILO 1264 (CAGCTCGGCGAAGGCCACTTGGCTGGG) ILO 1265 (TCGCTACCACAGTCGCAATCGTCGTCTCAAGG)	~400	5′ to 95 °C	30″ to 95 °C	30″ to 60 °C	1′ to 72 °C	10′ to 72 °C	[28,29]
	*Catl*	TviCatL1 (CGTCTCTGGCTCCGGTCAAAC) DTO155 (TTAAAGCTTCCACGAGTTCTTGATGATCCAGTA)	~177	5′ to 94 °C	30″ to 94 °C *	30″ to 65 °C	30″ to 72 °C	10′ to 72 °C	[20,30]
*T. evansi*	*ESAG*	ESAG 6/7F (ACATTCCAGCAGGAGTTGGAG) ESAG 6/7R (CACGTGAATCCTCAATTTTGT)	~237	4′ to 94 °C	1′ to 94 °C	1′ to 65 °C	30″ to 72 °C	5′ to 72 °C	[31,32]
*T. theileri*	*Catl*	TthCatL1 (CGTCTCTGGCTCCGGTCAAAC) DTO155 (TTAAAGCTTCCACGAGTTCTTGATGATCCAGTA)	~273	5′ to 95 °C	30″ to 95 °C *	30″ to 63 °C *	30″ to 72 °C *	10′ to 72 °C	[33,34]
*Babesia* sp.	*18S rRNA*	PIRO A (AATACCCAATCCTGACACAGGG) PIRO B (TTAAATACACGAATGCCCCCCCAAC)	~400	5′ to 94 °C	1′ to 94 °C	1′ to 61 °C	30″ to 72 °C	5′ to 72 °C	[18,35]
*B. bovis*	*rap-1*	BoF (CACGAGGAAGGAACTACCGATGTTGA) BoR (CCAAGGAGCTTCAACGTACGAGGTCA)	~356	5′ to 95 °C	1′ to 95 °C	1′ to 63 °C	30″ to 72 °C	7′ to 72 °C	[36,37]
*B. bigemina*	*hyp*	BilA (CATCTAATTTCTCTCCTACCCCTCC) BilB (CCTCGGCTTCAACTCTGATGCCAAAG)	~278	5′ to 95 °C	1′ to 95 °C	1′ to 60 °C	30″ to 72 °C	7′ to 72 °C	[38,39]
*A. marginale*	*msp5*	19A (GTTGTTCCTGGGGTACTCCTA) 19B (TGATCTGGTCAGCCCCAGCT)	~715	5′ to 94 °C	45″ to 94 °C	30″ to 64 °C	1′ to 72 °C	10′ to 72 °C	[40,41]

Legend: bp: base pairs; °C: degrees Celsius, ID: initial denaturation, D: denaturation, H: hybridization, E: extension, FE: final extension, ″: minutes, ′: seconds, *: 45 cycles.

**Table 2:** Distribution of prevalence and coinfections of *T. vivax*, *Babesia* spp. and *A. marginale* in the three sectors of Santa Cruz, Galapagos Island.

Infections	Bella Vista		Puerto Ayora		Santa Rosa		Total	
	Farm (n = 4)	Cattle (n = 40)	Farm (n = 2)	Cattle (n = 14)	Farm (n = 13)	Cattle (n = 116)	Farm (n = 19)	Cattle (n = 170)
	n (%)	n (%)	n (%)	n (%)	n (%)	n (%)	n (%)	n (%)
***T. vivax***	3 (75)	7 (17.5)	0	0	5 (38.5)	18 (15.5)	8 (42.1)	25 (14.7)
***B. bovis***	1 (25)	4 (10)	0	0	8 (61.5)	15 (12.9)	9 (47.4)	19 (11.2)
***B. bigemina***	3 (75)	4 (10)	2 (100)	2 (14.3)	10 (76.9)	19 (16.4)	15 (78.9)	25 (14.7)
***A. marginale***	4 (100)	23 (57.5)	2 (100)	10 (71.4)	13 (100)	81 (69.8)	19 (100)	114 (67.1)

**Table 3:** Details of coinfections of *T. vivax*, *B. bovis*, *B. bigemina* and *A. marginale* in cattle from Santa Cruz, Galapagos Island.

*T. vivax Da-PCR*	*B. bovis rap-1*-PCR	*B. bigemina hyp*-PCR	*A. marginale msp5*-PCR	Total (No. %)
**-**	-	-	-	42 (24.7)
**-**	-	-	+	75 (44.1)
**-**	-	+	-	3 (1.8)
**-**	-	+	+	11 (6.5)
**-**	+	-	-	3 (1.8)
**-**	+	-	+	3 (1.8)
**-**	+	+	-	1 (0.6)
**-**	+	+	+	7 (4.1)
**+**	-	-	-	7 (4.1)
**+**	-	-	+	12 (7.1)
**+**	-	+	+	1 (0.6)
**+**	+	-	+	3 (1.8)
**+**	+	+	+	2 (1.2)
				n = 170

**Table 4:** Presence and distribution of *T. vivax*, *B. bovis*, *B. bigemina* and *A. marginale* on farms on Santa Cruz, Galapagos Island.

*T. vivax Da-PCR*	*B. bovis rap-1*-PCR	*B. bigemina hyp*-PCR	*A. marginale msp5*-PCR	Total (No. %)
**-**	-	-	+	2 (10.5)
**-**	-	+	+	5 (26.3)
**-**	+	-	+	1 (5.3)
**-**	+	+	+	3 (15.8)
**+**	-	-	+	1 (5.3)
**+**	-	+	+	2 (10.5)
**+**	+	+	+	5 (26.3)
				n = 19

**Table 5:** Distribution of animals and positive results from molecular tests, based on zootechnical parameters of bovines analyzed in Santa Cruz, Galapagos Island.

Explanatory Variable	Modalities	Nº Animals Sampled (%)	*T. vivax* *Da*-PCR	*Babesia* spp. *18S rRNA*-PCR	*B. bovis* *rap-1*-PCR	*B. bigemina* *hyp*-PCR	*A. marginale msp5*-PCR
			n (%)	n (%)	n (%)	n (%)	n (%)
Sex	Male	12 (7.1)	1 (8.3)	6 (50)	3 (25)	6 (50)	9 (75)
	Female	158 (92.9)	24 (15.2)	28 (17.7)	16 (10.1)	19 (12)	105 (66.5)
Age	0–9 months	5 (3)	0	3 (60)	1 (20)	2 (40)	5 (100)
	10–18 months	16 (9.4)	4 (25)	10 (62.5)	8 (50)	9 (56.3)	15 (93.8)
	19–36 months	39 (22.9)	6 (15.4)	9 (23.1)	4 (10.3)	6 (15.4)	33 (84.6)
	>36 months	107 (62.9)	15 (14)	12 (11.2)	6 (5.6)	8 (7.5)	59 (55.1)
	ND	3 (1.8)	0	0	0	0	2 (66.7)
Breed	Crossbreed	30 (17.6)	3 (10)	10 (33.3)	5 (16.7)	8 (26.7)	28 (93.3)
	Holstein	28 (16.5)	3 (10.7)	2 (7.1)	0	2 (7.1)	16 (57.1)
	Brown Swiss	30 (17.6)	7 (23.3)	6 (20)	4 (13.3)	5 (16.7)	17 (56.7)
	Simmental	78 (45.9)	12 (15.4)	15 (19.2)	10 (12.8)	9 (11.5)	50 (64.1)
	ND	4 (2.4)	0	1 (25)	0	1 (25)	3 (75)

Legend: ND: not determined.

**Table 6:** Distribution of farms and positive results from molecular tests depending on sanitary management parameters used in farms of the Santa Cruz, Galapagos Island.

Explanatory Variable	Modalities	Total Farms (%)	*T. vivax* *Da*-PCR	*Babesia* spp. *18S rRNA*-PCR	*B. bovis* *rap-1*-PCR	*B. bigemina* *hyp*-PCR	*A. marginale msp5*-PCR
			n (%)	n (%)	n (%)	n (%)	n (%)
Production system	Meat	3 (15.8)	1 (33.3)	2 (66.7)	1 (33.3)	2 (66.7)	3 (100)
	Mixed	16 (84.2)	7 (43.8)	14 (87.5)	8 (50)	13 (81.3)	16 (100)
Livestock movement	No	8 (42.1)	5 (62.5)	7 (87.5)	6 (75)	7 (87.5)	8 (100)
	Yes	11 (57.9)	3 (27.3)	9 (81.8)	3 (27.3)	8 (72.7)	11 (100)
Separate sick cattle	No	5 (26.3)	2 (40)	4 (80)	2 (40)	4 (80)	5 (100)
	Yes	14 (84.2)	6 (42.9)	12 (85.7)	7 (50)	11 (78.6)	14 (100)
Change needles	No	9 (47.4)	4 (44.4)	8 (88.9)	3 (33.3)	7 (77.8)	9 (100)
	Yes	10 (57.9)	4 (40)	8 (80)	6 (60)	8 (80)	10 (100)
Pasture rotation	No	4 (21.1)	2 (50)	3 (75)	3 (75)	3 (75)	4 (100)
	Yes	15 (73.7)	6 (40)	13 (86.7)	6 (40)	12 (80)	15 (100)
Presence of vectors	Ticks	13 (76.5)	7 (53.8)	12 (92.3)	6 (46.2)	12 (92.3)	13(100)
	Ticks and flies	4 (23.5)	0	3 (75)	2 (50)	2 (50)	4 (100)
Presence of other domestic animals	No	6 (31.6)	1 (16.7)	5 (83.3)	2 (33.3)	5 (83.3)	6 (100)
	Yes	13 (68.4)	7 (53.9)	11 (84.6)	7 (53.8)	10 (76.9)	13 (100)
Presence of dead animals last year	No	12 (63.2)	5 (41.7)	11 (91.7)	7 (58.3)	10 (83.3)	12 (100)
	Yes	6 (31.6)	2 (33.3)	4 (66.7)	1 (16.7)	4 (66.7)	6 (100)
Presence of urine with blood	No	17 (89.5)	7 (41.2)	14 (82.4)	7 (41.2)	13 (76.5)	17 (100)
	Yes	2 (10.5)	1 (50)	2 (100)	2 (100)	2 (100)	2 (100)
Muscle tremors	No	18 (94.7)	8 (44.4)	15 (83.3)	8 (44.4)	14 (77.8)	18 (100)
	Yes	1 (5.3)	0	1 (100)	1 (100)	1 (100)	1 (100)

## Data Availability

Sample collection procedures were approved by the Ministerio del Ambiente, Agua y Transición Ecológica, MAE-DNB-CM-2017-0073.
